# Fungal‐Bacterial Interactions in Polymicrobial Infections: Hidden Threats

**DOI:** 10.1002/mbo3.70320

**Published:** 2026-05-28

**Authors:** Mohammad Javad Roustaye Gourabi, Masoud Kargar, Atefeh Kamali, Javad Yasbolaghi Sharahi

**Affiliations:** ^1^ Department of Microbiology, School of Medicine Shahid Beheshti University of Medical Sciences Tehran Iran; ^2^ Thalassemia and Hemoglobinopathy Research Center, Health Research Institute Ahvaz Jundishapur University of Medical Sciences Ahvaz Iran; ^3^ Department of Medical Microbiology (Bacteriology and Virology), Afzalipour School of Medicine Kerman University of Medical Sciences Kerman Iran; ^4^ Student Research Committee, Department of Microbiology, School of Medicine Shahid Beheshti University of Medical Sciences Tehran Iran

**Keywords:** antimicrobial resistance, biofilm viscoelasticity, diagnostic Challenges, fungal‐bacterial interactions, mixed‐species biofilms, therapeutic strategies

## Abstract

Polymicrobial infections involving fungi and bacteria represent a major and increasingly recognized clinical challenge, in which interkingdom interactions significantly amplify disease severity, antimicrobial resistance, and treatment failure. Rather than passive co‐existence, fungal–bacterial communities form highly coordinated systems driven by physical adhesion, quorum sensing, metabolic interdependence, and biofilm‐mediated structural reinforcement. These cooperative interactions, exemplified by pairs such as *Candida albicans*–*Staphylococcus aureus* and *Pseudomonas aeruginosa*–*Aspergillus fumigatus*, promote the development of treatment‐recalcitrant biofilms with enhanced immune evasion and multidrug tolerance. The global rise of multidrug‐resistant (MDR) and extensively drug‐resistant (XDR) pathogens has further intensified this burden, with polymicrobial biofilms now representing a post‐antibiotic clinical scenario in which therapeutic failure is driven not by individual resistant organisms but by emergent, cooperative resistance architectures. Conventional diagnostic approaches remain insufficient, as culture‐based methods frequently fail to capture the complexity of mixed microbial communities. Emerging technologies such as MALDI‐TOF mass spectrometry, metagenomic sequencing, and fluorescence in situ hybridization offer improved resolution but are not yet fully integrated into routine clinical practice. Therapeutically, increasing evidence indicates that monotherapy is inherently inadequate in polymicrobial infections due to the emergent nature of microbial cooperation. Effective management therefore requires combination strategies that simultaneously target multiple pathogens and their shared biofilm infrastructure. These include antibiotic–antifungal combinations, phage therapy, enzymatic and nanoparticle‐mediated biofilm disruption, metabolic interference, and host‐directed immunomodulation. Importantly, recent advances also highlight the role of biophysical properties such as biofilm viscoelasticity and matrix stiffness as critical and previously underappreciated therapeutic targets. This review uniquely integrates biochemical, biophysical, and therapeutic dimensions of polymicrobial infections into a unified systems‐level framework in which microbial cooperation is the central driver of pathogenesis, resistance, and treatment failure. Fungal–bacterial interactions are thereby positioned along a dynamic continuum from commensalism to pathogenesis, shaped by host susceptibility and environmental perturbations. Future progress will depend on interdisciplinary strategies combining multi‐omics technologies, precision diagnostics, and microbiome‐informed therapeutic design to effectively disrupt these complex microbial networks.

## 1 Introduction

Polymicrobial infections, defined as infections involving two or more microbial species such as bacteria, fungi or, viruses, have gained increasing clinical attention due to their association with heightened disease severity, reduced treatment efficacy and poorer patient outcomes (Krüger et al. 2019). Among these, fungal‐bacterial interactions represent a particularly challenging subset where synergistic relationships can dramatically exacerbate pathogenicity and antimicrobial resistance (AMR) (Nogueira et al. 2019). Recent studies have demonstrated that these cross‐kingdom interactions extend far beyond simple co‐existence, involving complex metabolic exchanges, quorum sensing (QS)‐mediated communication and coordinated immune evasion strategies (Todd and Peters 2019; Scheres and Krom 2016; Yasbolaghi Sharahi et al. 2025). For instance, the well‐documented synergy between *C. albicans* and *S. aureus* leads to enhanced biofilm formation in chronic wounds that is significantly more resistant to treatment than single‐species infections (Van Dyck 2021a). Similarly, the competitive yet co‐evolved relationship between *P. aeruginosa* and *A. fumigatus* in cystic fibrosis (CF) airways demonstrates how these interactions can shape disease progression (Keown et al. 2020).

The global escalation of MDR and XDR pathogens has further intensified the clinical burden of polymicrobial infections (Murray et al. 2022; Organization 2023; Roustaye Gourabi et al. 2026). MDR and XDR polymicrobial biofilms increasingly define a post‐antibiotic clinical landscape, where therapeutic failure is no longer attributable to individual resistant pathogens, but to emergent, cooperative resistance architectures that collectively evade all current antimicrobial classes. In high‐risk settings such as intensive care units, chronic wounds, device‐associated infections and immunocompromised patients, polymicrobial communities frequently harbor organisms exhibiting heterogeneous resistance phenotypes (De Waele and Boelens 2024; Magill et al. 2018). Within these structured microbial ecosystems, multiple mechanisms enhance antimicrobial tolerance. These include cross‐protection, horizontal gene transfer, metabolic cooperation, and biofilm‐mediated shielding (Sass et al. 2022; Zhao and Yu 2018). Fungal‐bacterial consortia such as *C. albicans* and *S. aureus* have demonstrated increased resistance and virulence when coexisting within polymicrobial biofilms (Van Dyck 2021a). These synergistic interactions not only compromise therapeutic efficacy but also accelerate the emergence and dissemination of resistance determinants (Orazi and O'Toole 2019; Ciofu et al. 2022). Polymicrobial biofilms constitute the central structural unit driving infection persistence and therapeutic failure (Van Dyck 2021a; Sass et al. 2022; Zhao and Yu 2018). Consequently, understanding polymicrobial dynamics in the context of MDR and XDR pathogens has become a critical priority in modern infectious disease research.

The self‐produced extracellular polymeric matrix not only provides mechanical stability but also creates protected niches where metabolic cooperation and quorum sensing‐mediated communication flourish, collectively amplifying pathogenic potential beyond what individual species can achieve (Todd and Peters 2019; Scheres and Krom 2016; Yasbolaghi Sharahi et al. 2025).

The clinical significance of these interactions is magnified by our incomplete understanding of their underlying mechanisms. Critical knowledge gaps persist regarding how fungal‐bacterial partnerships influence immune response modulation, contribute to biofilm resilience and develop shared AMR (Sass et al. 2022; Zhao and Yu 2018). Current diagnostic approaches remain largely inadequate for polymicrobial infections, as standard culture methods frequently fail to detect fastidious organisms within complex communities (Kullberg and Arendrup 2015). This diagnostic limitation often leads to inappropriate or delayed treatment, particularly in cases such as intra‐abdominal infections where *Enterobacteriaceae*‐*Candida* co‐infections are common but frequently undetected (Sartelli et al. 2024). Therapeutic strategies face similar challenges, as conventional antimicrobial regimens targeting single pathogens often prove ineffective against interdependent polymicrobial communities (Curtis et al. 2023).

Despite increasing recognition of fungal‐bacterial synergism, most current therapeutic and diagnostic paradigms remain largely pathogen‐centric, focusing on individual organisms rather than polymicrobial communities as integrated pathogenic units. Moreover, the mechanistic basis of interkingdom synergism, resistance amplification and biofilm‐associated tolerance in MDR/XDR settings remains incompletely elucidated.

This review synthesizes current knowledge of fungal–bacterial interactions in clinical infections. We focus on four main areas: molecular mechanisms of cross‐kingdom communication, diagnostic limitations and emerging technologies, resistance dynamics in polymicrobial biofilms, and novel therapeutic strategies targeting microbial communities as integrated systems.

## 2 Clinical Significance and Key Fungal‐Bacterial Pathogen Pairs

Fungal‐bacterial co‐infections, once often dismissed as contamination, are now recognized as a prevalent and clinically significant phenomenon. Modern diagnostics have revealed that active interactions between these kingdoms play a crucial role in exacerbating disease severity (Krüger et al. 2019). This section explores the most clinically relevant fungal‐bacterial partnerships.

### 2.1 *C. albicans* and *S. aureus*: A Model for Synergistic Biofilm Formation

The interaction between *C. albicans* and *S. aureus* is a well‐established model of polymicrobial synergy. These organisms frequently co‐colonize skin, mucosal surfaces, and medical devices. These pathogens frequently co‐colonize skin, mucosal surfaces, and medical devices. Their alliance is primarily mediated by *S. aureus* adhesion to the Als3p and Eap1p proteins on *C. albicans* hyphae, which is critical for stabilizing mixed biofilms (Nogueira et al. 2019; Scheres and Krom 2016; Khan et al. 2023; Alim et al. 2018). At a chemical level, *C. albicans*‐derived farnesol modulates *S. aureus* virulence by promoting toxin production and antibiotic resistance (Vila et al. 2019). This pathogen pair has been isolated from diverse biofilm‐associated infections, including periodontitis, denture stomatitis, burn wounds, and device‐related infections (Carolus et al. 2019). The clinical impact is severe. Epidemiological data from a 7‐year study found that 26.3% of candidemia patients had a concurrent bacterial infection, with such co‐infections leading to higher rates of septic shock, longer ICU stays, and significantly increased mortality (59% vs. 34.9%) (Zhong et al. 2022). ICU data modeling further indicates that co‐colonization increases the risk of *S. aureus* bacteremia (Hurley 2023). These observations are corroborated by animal models, where co‐infection results in greater disease severity, enhanced biofilm formation, and reduced antibiotic efficacy (Hu et al. 2021). Consequently, these interactions often necessitate medical device removal, posing significant risks for vulnerable patients (Todd and Peters 2019). In summary, the *C. albicans*–*S. aureus* interaction represents a well‐characterized model of synergistic polymicrobial biofilm formation, driven by combined physical adhesion and chemical signaling mechanisms, with important clinical implications for treatment outcomes.

### 2.2 *P. aeruginosa* and *A. fumigatus*: Antagonism and Co‐Existence in the CF Lung

In CF airways, the interaction between *P. aeruginosa* and *A. fumigatus* is highly complex. It involves both antagonistic and stimulatory effects that shape disease progression. *P. aeruginosa* employs a multi‐faceted attack, using its Las and Rhl QS systems to regulate the production of antifungal phenazines like pyocyanin to induce oxidative stress in the fungus (Keown et al. 2020; Bastos et al. 2022). It further competes for essential iron by secreting siderophores such as pyoverdine and pyochelin (6, 19). In response, *A. fumigatus* produces gliotoxin and undergoes phenotypic adaptations to resist bacterial suppression (Keown et al. 2020; Zhao and Yu 2018). Paradoxically, some interactions demonstrate stimulatory effects, as certain *Pseudomonas*‐derived volatiles can enhance fungal growth while the fungus may stimulate bacterial virulence factors like elastase production (Zhao and Yu 2018). These intricate dynamics likely explain the worse clinical outcomes observed in co‐infected CF patients, who typically experience more severe symptoms, chronic inflammation, and accelerated lung function decline compared to those with single‐pathogen infections (Sass et al. 2022).

This interaction highlights the complexity of polymicrobial dynamics, where antagonistic mechanisms can paradoxically contribute to sustained inflammation and disease progression.

### 2.3 *Enterobacteriaceae* and *Candida* spp: Synergy in Abdominal and Urinary Tract Infections

In the niches of abdominal and urinary tracts, *Candida* species frequently coexist with members of the Enterobacteriaceae family (e.g., *Escherichia coli*, *Klebsiella pneumoniae*). This co‐occurrence is particularly common in post‐surgical cases, intestinal perforations, and catheter‐associated urinary tract infections in immunocompromised or critically ill patients (Kullberg and Arendrup 2015; Sartelli et al. 2024; Roustaye Gourabi et al. 2025). While experimental models show some variability likely due to strain differences, clear synergistic effects have been documented. These include increased mortality in co‐infection models and *Candida*‐mediated creation of anaerobic microenvironments that promote bacterial persistence (Novy et al. 2023; Valentine et al. 2019). Clinical data strongly support these findings, showing that fungal‐bacterial co‐infections correlate with poorer treatment responses and prolonged catheterization needs (Sartelli et al. 2024). The synergistic relationship extends beyond *Candida*, with studies demonstrating that *A. fumigatus*‐*K. pneumoniae* interactions can also exacerbate infections and worsen patient prognosis, likely through enhanced inflammatory responses and microbial invasion (Curtis et al. 2023).

These interactions are primarily driven by metabolic and microenvironmental factors that facilitate persistence and therapeutic resistance. By altering the local pH and oxygen tension, Candida creates a favorable microhabitat for bacterial persistence, leading to chronic, difficult‐to‐eradicate infections that defy standard antimicrobial regimens, underscoring the importance of the infection microenvironment. A comparative summary of the molecular mechanisms, clinical impacts, and therapeutic challenges associated with these key pathogen pairs is provided in Table 3.

### 2.4 Case Studies: Clinical and Experimental Evidence of Polymicrobial Infection Dynamics

**Figure 1:**
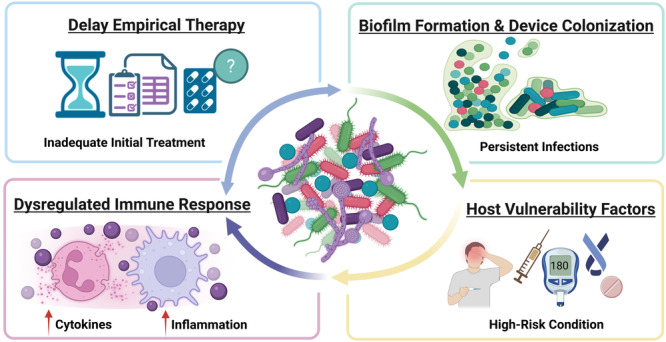
Integrated clinical and host determinants driving polymicrobial infection outcomes. Polymicrobial infections are governed by interconnected clinical, microbial, and host‐related factors rather than single‐pathogen effects. Delayed or inadequate empirical therapy increases the risk of early treatment failure, while biofilm formation on tissues or medical devices promotes persistence and antimicrobial tolerance. Concurrently, dysregulated host immune responses amplify inflammation and tissue damage, and underlying host vulnerability factors, including diabetes, immunosuppression, and invasive procedures, further predispose patients to severe disease. These elements form a reinforcing cycle that drives infection progression and worsens clinical outcomes.

To complement mechanistic insights, clinical evidence highlights that polymicrobial infections are driven by interacting biological and clinical factors rather than isolated microbial effects. Clinical and experimental case studies increasingly demonstrate that polymicrobial infections represent structured biological systems rather than incidental coexistence of microorganisms. These findings highlight that polymicrobial infection outcomes are governed by interconnected clinical and host‐related drivers, as summarized in Figure 1. Across diverse anatomical sites and patient populations, interactions between fungi and bacteria, or among multiple bacterial species, have been associated with altered virulence, therapeutic failure, and increased mortality (Mariani and Galvan 2023; Klotz et al. 2007; Peters and Noverr 2013).

In multicenter surveillance studies of candidemia in the United States, approximately 10% to 25% of cases were reported as polymicrobial bloodstream infections, frequently involving *Candida* spp. alongside Gram‐positive cocci such as *Staphylococcus epidermidis* and *Enterococcus faecalis*, or Gram‐negative bacilli including *K. pneumoniae* and *P. aeruginosa* (Klotz et al. 2007; Zhang 2020). These patients often exhibited delayed or initially inadequate empirical antimicrobial therapy, reflecting diagnostic uncertainty and the limitations of culture‐based identification strategies (Zhang 2020; Marra et al. 2005). The coexistence of bacteremia with candidemia challenges the traditional monomicrobial framework of bloodstream infection management and frequently necessitates broader‐spectrum and/or combination antimicrobial regimens (Klotz et al. 2007; Zhang 2020).

Device‐ and surgery‐associated infections further illustrate the structural resilience of polymicrobial systems. Case reports of persistent Candida glabrata candidemia resolving only after prosthetic mesh removal highlight the central role of foreign‐body‐associated biofilms in therapeutic failure (Etti et al. 2024). Experimental work on mixed *S. aureus*‐*C. albicans* biofilms demonstrates increased biomass, enhanced extracellular matrix production, and reduced antimicrobial susceptibility compared with monomicrobial biofilms (Mariani and Galvan 2023; Peters and Noverr 2013). These findings indicate that interspecies cooperation can generate emergent properties, particularly biofilm‐mediated tolerance, that cannot be predicted from single‐organism models (Mariani and Galvan 2023; Peters and Noverr 2013).

Experimental peritonitis models provide mechanistic insight into host‐microbe interactions during coinfection. In murine systems, coinfection with *C. albicans* and *S. aureus* resulted in substantially higher mortality than either organism alone, despite comparable initial pathogen burdens (Peters and Noverr 2013). The increased lethality correlated with amplified inflammatory mediators, including prostaglandin E_2_ and proinflammatory cytokines, suggesting that synergistic immune dysregulation, rather than simply additive microbial load, may drive disease severity (Peters and Noverr 2013). Similar patterns are reported in complex clinical settings such as post‐viral fungal infections complicated by secondary bacterial sepsis, where systemic inflammatory escalation appears central to poor outcomes (Meidani et al. 2024).

Host vulnerability consistently modulates polymicrobial infection dynamics. Burn injury, diabetes mellitus, immunosuppression, injection drug use, central venous catheterization, and CF create ecological niches characterized by tissue damage, impaired immunity, and frequent antimicrobial exposure (Mariani and Galvan 2023; Zhang 2020; Marra et al. 2005; Meidani et al. 2024). These conditions facilitate microbial coexistence, selection of multidrug‐resistant organisms, and persistent colonization. In CF airways, for example, chronic colonization by *P. aeruginosa* may interact with fungal species such as *A. fumigatus*, contributing to sustained inflammation and structural lung damage in up to 60% of patients chronically infected with *P. aeruginosa* (Briard 2022).

Critically, these case studies reveal several recurring limitations in current clinical management. First, diagnostic workflows often detect a dominant pathogen while overlooking additional microbial contributors, potentially delaying optimized therapy (Zhang 2020; Marra et al. 2005; Etti et al. 2024). Second, empirical treatment strategies are frequently calibrated toward single‐pathogen paradigms, increasing the risk of inadequate early coverage (Zhang 2020; Marra et al. 2005). Third, biofilm‐associated and device‐related infections underscore the necessity of aggressive source control, which is sometimes delayed or underappreciated in polymicrobial contexts (Peters and Noverr 2013; Etti et al. 2024).

Collectively, these case studies support the view that polymicrobial infections function as biologically interactive ecosystems shaped by microbial cooperation, host susceptibility, and therapeutic pressures. The evidence challenges reductionist, single‐pathogen models and instead favors integrated strategies combining rapid diagnostics, appropriately broad antimicrobial therapy, source control, and, in selected contexts, consideration of host‐directed immunomodulation (Peters and Noverr 2013). Recognizing polymicrobial infections as dynamic systems rather than static collections of organisms represents a fundamental paradigm shift with direct implications for clinical trial design, diagnostic development, and therapeutic innovation (Mariani and Galvan 2023; Klotz et al. 2007; Peters and Noverr 2013). Representative clinical and experimental case studies are summarized in Table 1.

**Table 1:** Representative case studies of polymicrobial infections.

Study context	Pathogens involved	Key findings	Clinical implications	Reference
Burn ICU bloodstream infections	*P. aeruginosa* + co‐pathogens (including *C. albicans*)	Burn injury significantly more common in polymicrobial infections (42.9% versus 10.4%); delayed appropriate therapy (3.4 vs. 1.7 days); inadequate empiric treatment in 85.7% of polymicrobial cases; progression to septic shock more strongly associated with mortality in polymicrobial cases (OR 38.5 vs. 4.5)	Burn patients require high index of suspicion for polymicrobial involvement; broader empirical coverage warranted; rapid diagnostic methods essential	(Marra et al. (2005))
Polymicrobial skin and soft tissue infections	*S. aureus* + *C. albicans*/*P. aeruginosa*	Up to 70% of *S. aureus* SSTIs are polymicrobial; in *S. aureus*‐*C. albicans* co‐culture: upregulation of *S. aureus* virulence factors, increased beta‐lactam/vancomycin resistance genes, enhanced biofilm formation; *S. aureus*‐*P. aeruginosa* co‐infection selects for small‐colony variants with stable aminoglycoside resistance	Empirical therapy for chronic wounds should consider both bacterial and fungal coverage; spatial organization within biofilms influences antimicrobial susceptibility	(Mariani and Galvan (2023))
Multicenter candidemia surveillance (CDC EIP)	*Candida* spp. + *S. aureus*, *K. pneumoniae*, *E. faecalis*	17.6% of candidemia cases polymicrobial; 23% of polymicrobial cases involved ≥ 3 organisms; polymicrobial patients younger (54.3 vs. 60.7 years); injection drug use (RR 1.95), CVC (RR 1.33), LTCF residence (RR 1.4) as risk factors; shorter time from admission to positive culture (1 vs. 4 days)	IDU, CVC, and LTCF exposure should raise suspicion for polymicrobial candidemia; targeted prevention strategies needed	(Zhang (2020))
Persistent post‐surgical candidemia	*C. glabrata* + *E. coli*	19‐day persistent candidemia with 18 positive cultures despite triple antifungal therapy (anidulafungin + voriconazole + flucytosine); infection cleared only after surgical mesh removal; initial *E. coli* peritonitis created niche for subsequent fungal infection	Foreign body removal essential in biofilm‐associated polymicrobial infections; bacterial co‐infection may predispose to persistent fungal infection	(Etti et al. (2024))
Post‐COVID‐19 mucormycosis with bacterial superinfection	Mucorales + MDR Gram‐negative bacteria (*A. baumannii*, *E. coli*, *K. pneumoniae*)	63% diabetic; secondary infections: bacteremia (83.3% mortality), pneumonia (80% mortality); 0% mortality in UTI/cellulitis; MDR *A. baumannii* in 18.5%; average 12.7 days from COVID‐19 onset to secondary infection	Diabetic patients post‐COVID‐19 require close monitoring for secondary bacterial infections; bacteremia and pneumonia carry extremely high mortality; MDR pathogens common	(Meidani et al. (2024))
Multi‐center VA and university hospital analysis	*Candida* spp. + multiple bacteria (69% Gram‐positive, 31% Gram‐negative)	27% of 372 candidemia patients had polymicrobial infections (24% with bacteria, 3% with multiple Candida species); literature review of 1882 patients: 23% polymicrobial; *Candida* occurs in a milieu of ongoing bacteremia	Nearly 1 in 4 candidemic patients have concurrent bacterial infection; empirical therapy should consider broad coverage; polymicrobial involvement is rule rather than exception	(Klotz et al. (2007))
CF airway colonization	*A. fumigatus* + *P. aeruginosa*	Up to 60% of CF patients with chronic *P. aeruginosa* also colonized by *A. fumigatus*; ABPA in up to 15%; hyperinflammation in co‐colonized patients; complex balance of inhibitory and stimulatory interactions	Fungal colonization in CF patients requires active surveillance; co‐colonization drives sustained inflammation and lung damage	(Briard (2022))
Murine peritonitis coinfection model	*C. albicans* + *S. aureus*	60% mortality in coinfection versus 0% in monomicrobial; 100‐fold increase in IL‐6 and G‐CSF; synergistic PGE_2_ induction; physical association of *S. aureus* with *C. albicans* hyphae in tissues; indomethacin (COX inhibitor) provided 100% protection and reduced microbial burden by 5‐6 logs	Immune dysregulation (eicosanoid storm) drives mortality in polymicrobial sepsis; immunomodulation may be therapeutic; physical microbial interactions occur in vivo	(Peters and Noverr (2013))

## 3 Mechanisms of Fungal‐Bacterial Interactions

**Figure 2:**
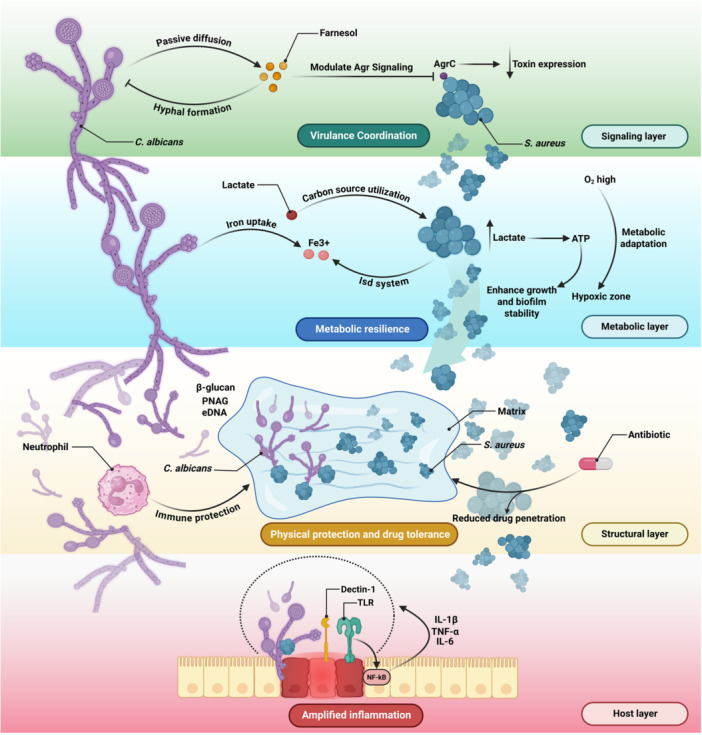
Multilayer mechanistic framework of fungal–bacterial synergy in polymicrobial infections. Fungal–bacterial interactions operate across multiple interconnected biological layers. At the signaling level, fungal‐derived molecules such as farnesol modulate bacterial quorum sensing systems (e.g., Agr), altering virulence expression. At the metabolic level, cross‐feeding (e.g., lactate utilization) and competition for key nutrients such as iron enhance microbial fitness and promote biofilm stability. Structurally, mixed‐species biofilms composed of EPS, β‐glucan, PNAG, and extracellular DNA provide mechanical protection and limit antimicrobial penetration. At the host interface, immune recognition through receptors such as Dectin‐1 and TLRs triggers amplified inflammatory responses. Together, these interconnected layers drive synergistic pathogenesis and therapeutic resistance.

The interactions between fungi and bacteria are governed by multiple molecular mechanisms. These interactions span signaling, metabolic, structural, and host‐response layers that collectively drive synergistic pathogenesis, as illustrated in Figure 2. These mechanisms can either enhance or inhibit pathogenicity depending on environmental context. A comparative overview of synergistic and antagonistic mechanisms is presented in Table 2.

**Table 2:** Comparative analysis of synergistic and antagonistic interactions between fungi and bacteria in polymicrobial infections.

Interaction type	Mechanism	Example	Clinical/Biological outcome
Synergistic	Co‐biofilm formation (fungal hyphae provide scaffolds for bacterial adhesion)	*C. albicans* and *S. aureus* in mixed biofilms	Enhanced antibiotic resistance (e.g., reduced susceptibility of *S. aureus* to vancomycin)
	Cross‐kingdom QS signaling (e.g., farnesol, AI‐2)	Farnesol from *C. albicans*, AI‐2 from bacteria	Modulation of growth, morphogenesis, and virulence in both microorganisms
	Immune evasion via neutrophil inactivation and biofilm shielding	Neutrophil dysfunction around mature *C. albicans* biofilms	Persistence of infection and chronic colonization
	Environmental pH alteration and stimulation of bacterial toxin production	Upregulation of α‐toxin production by *S. aureus* in the presence of *C. albicans*	Increased mortality and systemic infection severity
Antagonistic	Production of antibacterial metabolites (e.g., phenazines)	Phenazine secretion by *P. aeruginosa* against *C. albicans*	Inhibition of fungal growth and hyphal transformation
	Nutrient competition (iron and oxygen scavenging)	*C. albicans* secretes proteins that impair iron uptake by *P. aeruginosa*	Reduction in bacterial virulence
	Morphological inhibition of fungal pathogenic forms	3OC12‐HSL from *P. aeruginosa* prevents hyphal formation in *C. albicans*	Suppression of fungal invasiveness without affecting its overall growth
	Antibacterial fungal compounds	Gliotoxins and isocyanides from *A. fumigatus* against *P. aeruginosa*	Inhibition of bacterial biofilm formation and disruption of metabolic activity

**Table 3:** Clinically significant fungal‐bacterial pathogen pairs: molecular mechanisms, clinical consequences, and therapeutic implications.

Interaction type	Molecular mechanisms	Clinical consequences	Diagnostic challenges	Therapeutic implications	reference
*C. albicans* ‐ *S. aureus*	Adhesion of *S. aureus* to *C. albicans* hyphal proteins Als3p and Eap1p (stabilizing mixed biofilms) Cross‐kingdom QS (farnesol, AI‐2) β‐glucan–rich fungal matrix protecting bacteria Neutrophil inactivation and phagocytic escape	Highly drug‐resistant biofilms (e.g., reduced *S. aureus* susceptibility to vancomycin) Increased septic shock, ICU stays, and mortality (59% vs. 34.9%) Frequent need for medical device removal	Standard cultures may miss one or both partners within biofilms Complex matrices hinder microbial recovery Requires MALDI‐TOF MS, metagenomics, or FISH for accurate detection	Combination antibiotic–antifungal therapy (e.g., echinocandins + polymyxins) Biofilm‐disrupting enzymes (DNase I, Dispersin B) Silver/chitosan nanoparticles or phage therapy	(Nogueira et al. (2019); Todd and Peters (2019); Van Dyck (2021a); Khan et al. (2023); Alim et al. (2018); Vila et al. (2019); Carolus et al. (2019); Zhong et al. (2022); Hurley (2023); Hu et al. (2021); Wang et al. (2024); Todd (2019b); Kean et al. (2017); Kovács and Majoros (2020); Kunnath et al. (2024); Van Dyck (2021b); Tan et al. (2020); Yu et al. (2016); Jasim (2020); Shehabeldine et al. (2022); Kahl et al. (2023); Mahmud et al. (2024); Rahimi‐Midani et al. (2021); Manohar et al. (2024); Plumet et al. (2022); Osman et al. (2023); Hibstu et al. (2022); Kaplan et al. (2024); Wille and Coenye (2020); Elshinawy et al. (2018))
*P. aeruginosa* ‐ *A. fumigatus*	Phenazine production (pyocyanin) and siderophores (pyoverdine, pyochelin) for iron competition and oxidative stress Las and Rhl QS systems Fungal gliotoxin and isocyanides inhibiting bacteria Paradoxical stimulation of bacterial elastase or fungal growth	In CF lungs: chronic inflammation, accelerated lung function decline, more severe symptoms Fungal phenotypic adaptations to withstand bacterial antagonism	*P. aeruginosa* can suppress *A. fumigatus* growth in culture, causing false negatives Requires metagenomic sequencing or FISH‐CLSM for spatial biofilm analysis	Combination therapies (e.g., caspofungin + polymyxins) Nanoparticle‐based approaches (mesoporous silica + fluoroquinolones/metal ions) QS or metabolic interference strategies	(Keown et al. (2020); Sass et al. (2022); Zhao and Yu (2018); Bastos et al. (2022); MacAlpine et al. (2023a); Sass et al. (2019), 2018; Nazik et al. (2020); Briard et al. (2016); Lew et al. (2025); MacAlpine et al. (2023b); Danhof et al. (2016); Roy et al. (2018); Hattab et al. (2022); Fortes et al. (2023); Hussein et al. (2022); Shehabeldine et al. (2022); Álvarez et al. 2021; García et al. (2021); Afrasiabi and Partoazar (2024); Mohanta et al. (2022); Islam et al. (2025); Xie et al. (2025); Elshinawy et al. (2018); Bharathi and Lee (2025))
*Candida* ‐ *Enterobacteriaceae*	Environmental pH alteration and anaerobic niche creation by *Candida* Metabolic cross‐feeding and signaling exchanges Enhanced biofilm stability via shared extracellular matrices	More severe intra‐abdominal and urinary tract infections Increased mortality and prolonged catheterization Poorer response to standard treatments	Routine cultures often fail to detect these co‐infections Requires MALDI‐TOF MS, metagenomics, or spatial biofilm analyses for reliable identification	Targeted combination therapy Use of QS or metabolic inhibitors to disrupt cooperative interactions Nanotechnology or phage therapy to enhance drug penetration and efficacy	(Kullberg and Arendrup (2015); Sartelli et al. (2024); Curtis et al. (2023); Novy et al. (2023); Valentine et al. (2019); Roy et al. (2018); Fortes et al. (2023); Hussein et al. (2022); Zeidler et al. (2013); Álvarez et al. 2021; García et al. (2021); Afrasiabi and Partoazar (2024); Mohanta et al. (2022); Islam et al. (2025); Xie et al. (2025); Nithyanand et al. (2025); Pan et al. (2024); Eichelberger and Cassat (2021a), 2021b; Didehdar et al. (2022); Jastrzębowska and Gabriel (2015); Ahmad et al. (2025); Bharathi and Lee (2025))

### 3.1 Synergistic Interactions

Synergistic interactions lead to enhanced pathogenicity, increased AMR, and worse clinical outcomes.

#### 3.1.1 Enhanced Biofilm Formation and Antimicrobial Resistance

Mixed‐species biofilms are a central feature of synergistic interactions. They provide structural stability and increased protection against antimicrobial agents. In *C. albicans* and *S. aureus* biofilms, the fungal matrix, particularly its β‐glucan content, protects the bacterium from vancomycin. Inhibiting glucan synthesis disrupts this protective symbiosis (Wang et al. 2024; Soe et al. 2021). Conversely, the protective role can be bacterial‐driven; in *Streptococcus mutans* and *C. albicans* co‐biofilms, bacterial α‐glucan enhances the fungus's antifungal resistance (Nett and Andes 2020). Similarly, the *C. albicans* matrix can increase antibiotic resistance in *E. coli* within polymicrobial communities.

In summary, the reciprocal protection within mixed biofilms, mediated by shared matrix components, creates a formidable physical barrier that significantly enhances community‐wide antimicrobial tolerance, rendering standard monotherapies ineffective.

#### 3.1.2 Cross‐Kingdom Signaling

Interdomain communication via QS molecules is a key mechanism of synergy. Fungal‐derived farnesol and bacterial‐derived autoinducer‐2 (AI‐2) reciprocally regulate virulence gene expression, metabolic pathways, and morphological shifts. Farnesol from *C. albicans* can inhibit some bacteria while altering physiological properties in others, such as increasing *S. aureus* α‐toxin production and antibiotic resistance (MacAlpine et al. 2023a; Todd 2019a, 2019b). Bacterial AI‐2, in turn, can induce or suppress fungal germination and hyphal transformation, critically influencing biofilm development and pathogenicity (Lee et al. 2016; Bamford et al. 2009; Kostoulias et al. 2016).

This illustrates that cross‐kingdom signaling is not merely communication but a sophisticated tool for co‐regulation, enabling fungi and bacteria to fine‐tune each other's virulence and lifestyle in a context‐dependent manner, which complicates predicting infection outcomes.

#### 3.1.3 Joint Immune Evasion

Polymicrobial communities collaboratively evade host defenses. Although neutrophils detect mature *C. albicans* biofilms, they often remain inactive at the biofilm periphery, failing to mount an effective reactive oxygen species (ROS) response (Gulati and Nobile 2016; Juszczak et al. 2025). Furthermore, fungal hyphae play a crucial role in phagocytic escape. Their elongated structure allows them to physically penetrate epithelial layers and rupture phagocyte membranes, facilitating the survival and dissemination of both themselves and co‐infecting bacteria (Gulati and Nobile 2016; Zhao et al. 2022). This collective immune suppression is a major factor in infection chronicity.

Collectively, this demonstrates a division of labor in immune evasion, where the physical robustness of fungal hyphae provides an escape route for both pathogens, while the biofilm structure collectively neutralizes neutrophil attacks, leading to persistent infections.

#### 3.1.4 Induction of Bacterial Antibiotic Resistance

Beyond providing physical protection, fungi actively induce genetic and phenotypic changes in bacteria that lead to enhanced antibiotic resistance (Mousavi et al. 2025). A key mechanism involves the impairment of antibiotic penetration through the fungal extracellular matrix (EPS). The *C. albicans* EPS, particularly mannan and β‐glucans, creates a diffusion barrier that significantly limits vancomycin penetration, protecting embedded *S. aureus* cells (Kean et al. 2017).

Furthermore, fungal metabolites directly modulate bacterial resistance pathways. Farnesol secreted by *C. albicans* upregulates efflux pumps and stress response systems in S. aureus, leading to enhanced multidrug tolerance (Kovács and Majoros 2020). This signaling molecule can also alter cell membrane properties, further reducing antimicrobial susceptibility.

Additionally, the fungal microenvironment promotes the formation of bacterial persister cells. Within mixed biofilms, *C. albicans* induces a slow‐growing, metabolically dormant state in *S. aureus* that exhibits intrinsic tolerance to conventional antibiotics, contributing to chronic and relapsing infections (Kunnath et al. 2024).

In conclusion, fungi employ a multi‐pronged strategy to induce bacterial antibiotic resistance, combining physical barrier formation with active modulation of bacterial physiology. These mechanisms, including impaired drug diffusion, upregulation of efflux systems, and promotion of persister phenotypes, collectively contribute to the remarkable resilience of polymicrobial infections against conventional antimicrobial therapies.

### 3.2 Antagonistic Interactions

Antagonistic interactions involve microbial warfare, where one organism suppresses the growth or virulence of the other.

#### 3.2.1 Bacterial Inhibition of Fungal Growth and Morphology

Bacteria can directly suppress fungal virulence. *P. aeruginosa* secretes QS molecules like 3‐oxo‐C12‐homoserine lactone (3OC12HSL), which specifically inhibits the critical yeast‐to‐hyphal transition in *C. albicans* without affecting its overall growth, thereby reducing its pathogenicity (Hogan et al. 2004; Coquant et al. 2022). *P. aeruginosa* also employs phenazines (e.g., pyocyanin) and iron competition via siderophores to suppress the growth of *C. albicans* and *A. fumigatus* (Sass et al. 2019, 2018; Nazik et al. 2020; Briard et al. 2016). Notably, phenazines like pyocyanin may play a protective role against candidiasis in the lung by inhibiting fungal growth and modulating host immune responses (Lew et al. 2025).

In conclusion, bacterial antagonism often employs a targeted strategy, disarming key fungal virulence mechanisms like hyphal formation rather than outright killing, which can paradoxically modulate disease presentation without eliminating the fungal burden.

#### 3.2.2 Fungal Disruption of Bacterial Virulence

Fungi are not passive targets and have evolved countermeasures. *C. albicans* secretes farnesol, which inhibits the *Pseudomonas* quinolone signal pathway, and proteins that disrupt bacterial iron uptake, reducing *P. aeruginosa* pathogenicity. Animal studies show that administering these fungal proteins can prevent *P. aeruginosa* infection in mice, an effect neutralized by iron supplementation (Sass et al. 2019, 2018; Nazik et al. 2020; Briard et al. 2016; MacAlpine et al. 2023b). Similarly, *A. fumigatus* produces antibacterial compounds like gliotoxin and isocyanides, which induce oxidative stress and disrupt metabolic pathways in *P. aeruginosa*, inhibiting its growth and biofilm formation (Sass et al. 2019, 2018; Nazik et al. 2020; Briard et al. 2016).

This reveals that fungi actively engage in microbial warfare, deploying specific molecules to disrupt critical bacterial functions such as QS and iron homeostasis, highlighting an ongoing arms race that shapes the dynamics of co‐infection.

#### 3.2.3 Metabolic Cross‐Feeding and Niche Modification

*C. albicans* possesses a remarkable ability to modify its local environment, creating favorable niches for itself and facilitating co‐colonization with bacterial partners. A key mechanism is environmental acidification through proton extrusion, which reduces the pH of the surrounding medium to levels compatible with host tissues (Danhof et al. 2016). This acidification is often accompanied by increased CO_2_ concentrations, which can further enhance *C. albicans* adhesion to epithelial cells and simultaneously generate conditions that favor the proliferation of acidophilic bacterial species (Pentland et al. 2021). Beyond mere co‐existence, these interactions involve active metabolic cooperation. Within multispecies biofilms, metabolic cross‐feeding is a critical driver of community stability, where waste products or secreted metabolites from one species serve as nutrient sources or signals for another (Joshi et al. 2021). This reciprocal exchange facilitates bacterial growth in the fungal vicinity, particularly when bacteria efficiently utilize fungal‐derived metabolites, thereby reinforcing the composite biofilm structure and resilience (Sharma et al. 2023; Kost et al. 2023). This metabolic dialog is often governed by sophisticated QS mechanisms. *C. albicans* produces and responds to signaling molecules such as farnesol and tyrosol, which regulate its own morphology, biofilm development, and virulence (Kovács and Majoros 2020). Conversely, bacterial autoinducers can modulate fungal behavior, including growth dynamics and hyphal morphogenesis (Jothi et al. 2021).

In summary, the metabolic interplay between fungi and bacteria extends beyond simple competition to include sophisticated niche modification and cross‐feeding. This creates a mutually reinforced metabolic synergy where environmental changes by one partner (e.g., acidification by *Candida*) can create a favorable niche for the other, ultimately leading to a more stable and resilient polymicrobial community that is adept at co‐colonization and pathogenesis.

### 3.3 Biophysical and Mechanical Determinants of Polymicrobial Biofilms

Beyond biochemical signaling and metabolic interactions, the biophysical properties of polymicrobial biofilms play a critical role in their persistence and therapeutic recalcitrance. The EPS matrix confers viscoelastic behavior, a complex mechanical phenotype combining both elastic (solid‐like) and viscous (fluid‐like) responses, that governs biofilm integrity and resistance to mechanical and chemical perturbations. Viscoelasticity has been recognized as a key determinant of biofilm physiology and drug tolerance (Shaw et al. 2004).

Viscoelasticity describes materials that deform elastically under low stress but flow viscously under sustained stress. Biofilms, particularly mixed communities of fungi and bacteria, exhibit unique rheological signatures distinct from single‐species biofilms due to heterogeneous EPS composition. Fungal structures such as *C. albicans* hyphae act as micromechanical reinforcements, increasing the elastic modulus and structural robustness of mixed biofilms. Higher elastic modulus correlates with increased resistance to shear forces typical of physiological environments including wound exudate and respiratory tracts. Mechanical robustness allows mixed biofilms to persist on indwelling devices and mucosal surfaces where fluid shear would otherwise dislodge less cohesive communities (Banerjee et al. 2011; Petrova and Sauer 2012).

Matrix stiffness, quantified by rheometry or atomic force microscopy (AFM), directly influences antimicrobial diffusion. Stiffer matrices present a physical barrier that retards penetration, leading to sub‐therapeutic local drug concentrations. For example, increased β‐glucan content in dual‐species *C. albicans*‐*S. aureus* biofilms correlates with enhanced resistance to antibiotic penetration. Viscoelastic properties also modulate microbial physiology through mechanotransduction, where mechanical cues within the biofilm matrix can alter gene expression and stress responses, leading to increased persistence and tolerance (Tallawi et al. 2017).

Standard static culture models inadequately replicate the biophysical environment of polymicrobial communities. Microfluidic platforms, coupled with high‐resolution imaging (e.g., confocal laser scanning microscopy, CLSM), have emerged as powerful tools to observe biofilm mechanics, microbial interactions, and drug responses in real time under controlled shear, nutrient gradients, and flow conditions. Recent real‐time polymicrobial imaging studies have demonstrated that mixed‐species biofilms exhibit dynamic spatial reorganization, time‐dependent mechanical adaptation, and flow‐responsive structural remodeling, highlighting the critical role of physical microenvironments in shaping interkingdom interactions. Time‐lapse microscopy in microfluidic systems enables quantification of adhesion kinetics, biofilm consolidation, and differential responses to antimicrobial exposure, revealing that conventional antibiotics demonstrate delayed killing kinetics in polymicrobial biofilms compared to planktonic cultures (Richter et al. 2007; Kim et al. 2012; Straub et al. 2020).

Mechanically targeted therapies aim to disrupt the structural integrity of biofilms to improve antimicrobial access. Enzymatic matrix disruption using DNase I (targeting extracellular DNA), dispersin B (targeting PNAG), or alginate lyase has shown synergy with antibiotics in reducing mechanical stability and enhancing drug penetration. Ultrasound‐assisted therapy leverages low‐frequency acoustic waves to transiently disrupt EPS structure, improving antimicrobial delivery, particularly in device‐associated biofilms. Photothermal therapy, using light‐activated nanoparticles such as gold nanoshells, reduces matrix cohesion locally and enhances antibiotic susceptibility without systemic toxicity. Additionally, magnetic nanoparticles can mechanically perturb biofilms under oscillating magnetic fields, further increasing antimicrobial penetration and efficacy (Kumar et al. 2021).

Integrating biophysical perspectives into polymicrobial infection research reveals that the mechanical foundation of biofilm structure is as critical as biochemical signaling in determining treatment outcomes. By targeting both structural integrity and microbial viability, mechano‐therapeutic strategies represent a promising frontier in combating recalcitrant polymicrobial infections.

## 4 Diagnostic Challenges

The accurate diagnosis of polymicrobial infections is fraught with difficulties, primarily due to the limitations of conventional culture‐based methods and the complex nature of microbial interactions.

### 4.1 Limitations of Conventional Culture Methods

Standard culture methods significantly underestimate microbial diversity in polymicrobial infections. Fastidious organisms often fail to grow under routine laboratory conditions (Latgé and Chamilos 2019; Eriksen et al. 2023). Furthermore, antagonistic interactions can suppress the growth of one partner; for instance, *P. aeruginosa* secretes QS molecules and antifungal metabolites (e.g., phenazines, pyrrolnitrin) that actively inhibit the growth and biofilm formation of *A. fumigatus* in culture, leading to its false‐negative reporting (Sass et al. 2019; Rozaliyani et al. 2023; Selegato and Castro‐Gamboa 2023). Within mixed biofilms, the protective matrix and altered microenvironments (pH, oxygen, nutrients) further reduce microbial cultivability and increase antibiotic tolerance, making recovery and isolation of individual species challenging (Roy et al. 2018).

In essence, conventional culture methods provide a distorted and incomplete picture of polymicrobial infections, critically underestimating microbial diversity and missing key pathogens due to both biological fastidiousness and intermicrobial warfare, which directly contributes to inappropriate or delayed treatment.

### 4.2 Advanced Molecular and Culture‐Independent Techniques

To overcome these limitations, culture‐independent diagnostic techniques are essential.

#### 4.2.1 MALDI‐TOF Mass Spectrometry

MALDI‐TOF MS is a rapid and accurate tool for microbial identification. Its operation is based on the ionization of microbial cells with short laser pulses to generate a unique species‐specific spectral fingerprint (Sandrin and Demirev 2018; Jang and Kim 2018; Franco‐Duarte et al. 2019). It has found widespread use in identifying *Candida* species with high accuracy and shows promise in detecting antifungal resistance in yeasts, though its performance is more limited for molds (Lau 2021).

This makes MALDI‐TOF an excellent tool for rapid identification of cultivable pathogens in a polymicrobial context, yet its utility is constrained by its inability to detect uncultivable organisms or resolve complex communities within a single sample.

#### 4.2.2 Phylogenetic Marker‐Based Methods (Amplicon Sequencing)

The development of PCR and sequencing technologies, targeting phylogenetic markers like the 16S rRNA gene for bacteria and the ITS region for fungi, revolutionized microbial community analysis. This metabarcoding approach allows for the identification of a wide range of cultivable and uncultivable species (Berg et al. 2020; Maritz et al. 2017; Purahong et al. 2016). However, while powerful for determining composition, these methods provide limited information on the metabolic state or activity of the detected microbes (Berg et al. 2020).

Thus, while amplicon sequencing provides high‐sensitivity taxonomic identification of microbial community composition, it fails to reveal functional activity or metabolic state, leaving a critical gap in understanding the infection dynamics.

#### 4.2.3 Shotgun Metagenomics

Shotgun metagenomics sequences the entire DNA content of a sample, providing a comprehensive view of the microbiome and the resistome (the collection of AMR genes). This powerful method can identify all microorganisms and detect rare species but requires higher costs and sophisticated bioinformatics analysis (Barraud et al. 2019; Quince et al. 2017).

Therefore, shotgun metagenomics represents a significant leap forward by providing both taxonomic and functional genetic data, but its high cost and computational demands currently limit its routine clinical application.

### 4.3 Techniques for Spatial Analysis in Biofilms

Understanding the spatial organization of polymicrobial biofilms is crucial, as structure dictates function and interaction. While NGS and RNA‐seq reveal taxonomic and functional profiles, they destroy spatial context. Fluorescence in situ hybridization (FISH) overcomes this by using fluorescently‐labeled DNA/RNA probes to visually locate specific microorganisms within an intact biofilm. Advanced variations like multicolor FISH enable simultaneous examination of multiple targets (Quince et al. 2017; Barbosa et al. 2023). When combined with confocal laser scanning microscopy (CLSM), FISH enables high‐resolution 3D reconstruction of the biofilm architecture, providing invaluable insights into microbial interactions (Mhade and Kaushik 2023).

In summary, techniques like FISH‐CLSM are indispensable for moving beyond mere species lists to understanding the spatial ecology of polymicrobial infections, revealing how physical proximity and community structure drive pathogenicity and resistance.

### 4.4 The Path Forward: Integrated Diagnostic Approaches

No single method is sufficient. The most efficient strategy for diagnosing polymicrobial infections involves a synergistic combination of these advanced techniques: using MALDI‐TOF for rapid identification, amplicon sequencing or metagenomics for community profiling, and FISH/CLSM for contextual spatial analysis within biofilms.

Critically, the future of diagnosis lies not in seeking a single superior technology, but in developing integrated, pragmatic pipelines that leverage the strengths of each method. The major hurdle is no longer technical, but translational: transforming these powerful, often complex approaches into cost‐effective, standardized, and clinically accessible tools for routine practice to guide targeted therapy effectively.

## 5 Therapeutic Strategies and Challenges

**Figure 3:**
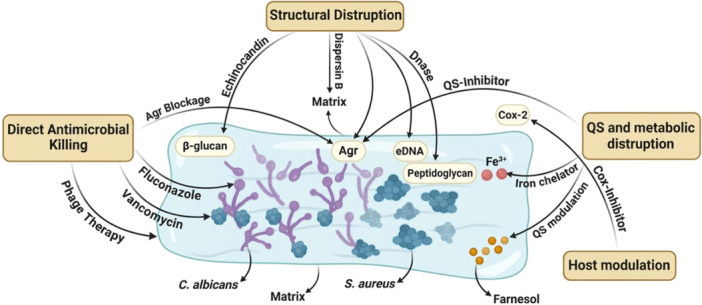
Mechanism‐guided combinatorial therapeutic framework for polymicrobial infections. Effective treatment of polymicrobial infections requires multi‐target strategies that disrupt microbial cooperation across biological layers. Direct antimicrobial agents reduce pathogen burden, while structural targeting approaches, including DNase, Dispersin B, and echinocandins, degrade biofilm integrity and enhance drug penetration. Metabolic and quorum sensing inhibitors interfere with interkingdom signaling and nutrient exchange, and host‐directed therapies modulate dysregulated inflammatory responses. Integration of these approaches through mechanism‐guided combinatorial regimens, supported by precision diagnostics, aims to overcome biofilm‐associated tolerance and improve therapeutic outcomes despite existing translational challenges.

The therapeutic failure observed in polymicrobial infections is not merely additive but represents an emergent property of multilayer microbial cooperation (Figure 2). The treatment of fungal‐bacterial polymicrobial infections is notoriously difficult, requiring strategies that overcome inherent resistance mechanisms and complex microbial interactions. Accordingly, effective management requires mechanism‐guided combinatorial strategies that simultaneously target microbial burden, biofilm structure, interkingdom signaling, and host responses, as illustrated in Figure 3.

### 5.1 Current Challenges and Limitations

Therapeutic failure in polymicrobial infections is not simply additive. It emerges from cooperative interactions within microbial communities.

#### 5.1.1 Biofilm‐Mediated Tolerance

The mixed biofilm matrix acts as a primary barrier, significantly limiting the penetration of both antibacterial and antifungal drugs, thereby facilitating pathogen survival and fostering recalcitrant infections (Rodrigues et al. 2019). This physical and chemical barrier not only reduces drug penetration but also creates heterogeneous microenvironments that promote persistent, dormant cell phenotypes, rendering standard pharmacokinetic principles largely ineffective against biofilm‐embedded communities.

#### 5.1.2 Antagonistic Drug Interactions

Therapeutic efficacy is further complicated by direct and indirect drug interactions. A prime example is the QS molecule N‐(3‐Oxododecanoyl)‐l‐homoserine lactone from *P. aeruginosa*, which can induce resistance in *Candida* to fluconazole by altering the ergosterol biosynthesis pathway and enhancing drug efflux (Bandara et al. 2020). Paradoxically, other studies show that *P. aeruginosa* can enhance fluconazole activity through iron sequestration, an effect reversed by iron supplementation (Hattab et al. 2022).

This apparent paradox underscores the critical context‐dependency of polymicrobial interactions, where outcomes are determined by specific environmental factors such as local concentration of signaling molecules, pH, iron availability, and the metabolic state of the pathogens. This complexity makes standardized treatment protocols particularly challenging and highlights the need for personalized therapeutic approaches. Therapeutic failure in polymicrobial infections is an emergent systems‐level property rather than the additive sum of individual resistance traits. Within polymicrobial biofilms, fungi and bacteria form interdependent networks in which physical shielding, metabolic cooperation, and coordinated stress responses collectively generate a higher‐order resistance phenotype. Consequently, monotherapies targeting single pathogens are intrinsically limited, as they fail to disrupt the ecological and functional integrity of the community. This provides a mechanistic justification for combination strategies, which are required not merely to broaden antimicrobial coverage, but to dismantle the cooperative architecture that underpins collective tolerance.

### 5.2 Conventional Antimicrobial Approaches and Limitations

#### 5.2.1 Antibiotic‐Antifungal Synergistic Combinations

Combination therapy is a promising strategy to enhance efficacy. Polymyxins show particular promise when paired with antifungals. Synergy has been reported with miconazole against Gram‐negative bacteria and yeasts (Xie et al. 2022), with caspofungin against *P. aeruginosa* and *K. pneumoniae* biofilms (Fortes et al. 2023; Hussein et al. 2022), and with echinocandins against Candida species, where cell wall disruption by the antifungal facilitates membrane attack by the antibiotic (Zeidler et al. 2013).

While these combinations show improved efficacy in vitro, their clinical translation remains limited by potential toxicity concerns and the lack of standardized guidelines for polymicrobial infections.

#### 5.2.2 Antimicrobial Peptides and Peptidomimetics

Antimicrobial peptidomimetics (e.g., TM8, RK758) represent a novel class of agents that disrupt microbial membranes. They exhibit strong synergistic effects with conventional antifungals against resistant yeast isolates, primarily by compromising cell membrane integrity (Mishra et al. 2025).

These broad‐spectrum agents offer advantages against polymicrobial infections but face challenges in stability, delivery, and potential host cytotoxicity that require further optimization.

### 5.3 Advanced Anti‐Biofilm Strategies

#### 5.3.1 Enzymatic Biofilm Disruption

Enzymes that degrade key biofilm components are effective anti‐biofilm strategies. DNase I targets extracellular DNA, while Dispersin B hydrolyzes poly‐N‐acetylglucosamine (PNAG). When co‐immobilized on chitosan nanoparticles, these enzymes achieve greater than 80% disruption of preformed biofilms (Van Dyck 2021b; Tan et al. 2020). DNase I, which targets extracellular DNA, can reduce *S. aureus* attachment to *C. albicans* biofilms at high concentrations (Kean et al. 2017).

The enzymatic approach represents a targeted strategy against specific biofilm matrix components, though enzyme stability and delivery remain key challenges for clinical application.

#### 5.3.2 d‐Amino Acids and Small Molecule Inhibitors

D‐amino acids (e.g., d‐tyrosine, d‐aspartic acid) inhibit biofilm formation and disrupt pre‐existing structures in various pathogens. Their antibiofilm effect is synergistically enhanced when combined with nanoparticles or other agents like lithium (Roy et al. 2018; Yu et al. 2016) (Jasim 2020).

These compounds work by interfering with protein synthesis and incorporation into cell walls, preventing proper biofilm maturation without exerting direct killing pressure that could select for resistance.

### 5.4 Innovative Nanotechnology Platforms

#### 5.4.1 Multifunctional Nanoparticle Systems

Nanoparticles offer a multifaceted platform for combating polymicrobial infections. Silver nanoparticles (AgNPs), especially when stabilized with chitosan, exhibit broad‐spectrum antimicrobial, antibiofilm, and antifungal activities (Shehabeldine et al. 2022). More sophisticated systems include mesoporous silica nanoparticles co‐loading antibiotics and metal ions, and core@shell nanostructures integrating drug delivery with nitric oxide release and photothermal therapy (Álvarez et al. 2021; García et al. 2021).

Nanotechnology provides unprecedented opportunities for targeted drug delivery and combination therapy, with the ability to penetrate biofilms and release payloads in response to specific environmental triggers.

#### 5.4.2 Mechanism of Nanotherapeutic Action

Metal‐based nanoparticles (e.g., Ag, ZnO, CuO, Fe_3_O_4_) exert their effects through multiple mechanisms: inducing lethal ROS production, disrupting cellular membranes, and downregulating key genes essential for EPS synthesis (e.g., *lasI*/*lasR* in *P. aeruginosa* and *icaA* in *S. aureus*), thereby destabilizing the biofilm architecture (Afrasiabi and Partoazar 2024; Mohanta et al. 2022; Islam et al. 2025; Xie et al. 2025).

The multi‐mechanistic action of nanomaterials reduces the likelihood of resistance development while simultaneously targeting both structural and functional aspects of polymicrobial communities.

### 5.5 Biological and Metabolic Intervention Strategies

#### 5.5.1 Precision Phage Therapy

Phage therapy offers a highly targeted strategy for polymicrobial infections. It selectively lyses bacterial partners without affecting fungal cells. The co‐habitation of pathogens like *C. albicans* with *S. aureus* or *P. aeruginosa* in biofilms significantly enhances antimicrobial tolerance and complicates treatment (Carolus et al. 2019; Kahl et al. 2023). Phages offer a precise solution by selectively lysing the bacterial partner without disrupting fungal cells or the surrounding host microbiota, simultaneously degrading the biofilm's structural integrity by targeting bacterial components of the EPS (Mahmud et al. 2024; Rahimi‐Midani et al. 2021).

The efficacy of phage therapy extends beyond simple bacterial killing. By significantly reducing bacterial density, phages enhance the penetration of concurrently administered antimicrobial agents into the biofilm and can restore susceptibility to these drugs, effectively reversing the tolerance facilitated by the polymicrobial environment (Manohar et al. 2024; Plumet et al. 2022).

Consequently, the most promising application involves combination therapy, where phages are used synergistically with antifungal agents. The phage‐mediated dismantling of the bacterial infrastructure and reduction in bacterial load dramatically improve the access and efficacy of antifungals (Osman et al. 2023; Hibstu et al. 2022). Furthermore, certain phages can inhibit critical bacterial virulence pathways, including the production of extracellular polysaccharides and QS signaling molecules, leading to the profound destabilization of the mixed biofilm matrix (Chegini et al. 2020).

Compelling evidence from in vitro studies and animal models demonstrates the success of this approach. The combination of anti‐*S. aureus* phages with antifungals (e.g., fluconazole, amphotericin B) has proven highly effective against dual‐species *Candida*‐*S. aureus* and *Candida*‐*Pseudomonas* biofilms, resulting in a significant reduction of microbial burden and improved survival rates (Hu et al. 2021; Lohse et al. 2020; Bonincontro et al. 2023). This targeted strategy highlights the potential of phage therapy as a cornerstone for next‐generation treatments against recalcitrant polymicrobial infections.

Phage therapy offers species‐specific targeting without disrupting beneficial microbiota, though phage resistance development and regulatory hurdles remain significant challenges.

#### 5.5.2 Metabolic Interference and Quorum Quenching

A sophisticated approach to disrupting polymicrobial infections involves metabolic interference, which aims to exploit and disrupt the essential metabolic crosstalk and shared pathways that underpin fungal‐bacterial synergy (Nithyanand et al. 2025). These interactions, crucial for energy acquisition, environmental adaptation, and community stability, present a novel set of targets for therapeutic intervention (Pan et al. 2024). A prime example is the metabolic interplay in *Candida*‐*staphylococcal* co‐infections, where *C. albicans* can break down host amino acids, releasing ammonia as a byproduct and shifting the local microenvironment toward alkalinity (Eichelberger and Cassat 2021a). This pH change acts as a potent environmental cue that stimulates the *S. aureus* Agr QS system, a master regulator of virulence and biofilm formation, thereby exacerbating infection severity (Eichelberger and Cassat 2021b). The strategy of metabolic interference involves designing agents that block this detrimental communication, potentially through inhibiting fungal amino acid catabolism to prevent ammonia production and the subsequent pH shift, developing targeted Agr inhibitors (e.g., synthetic QS inhibitors or small molecules) that block the bacterial response to the fungal signal, effectively deafening the bacterium to this interkingdom cue, or targeting other shared metabolic dependencies such as the competition for iron through siderophore systems or the exchange of essential nutrients and metabolites that sustain co‐growth within the biofilm (Didehdar et al. 2022; Jastrzębowska and Gabriel 2015). By precisely disrupting these metabolic conversations, this strategy seeks to collapse the cooperative infrastructure of the polymicrobial community, rendering it more susceptible to conventional antimicrobial agents and host defenses, ultimately moving beyond merely killing microbes to intelligently manipulating their behavior and interactions (Nithyanand et al. 2025; Ahmad et al. 2025).

## 6 The Polymicrobial Continuum: From Commensalism to Pathogenesis

Within the healthy microbiome, fungal–bacterial interactions are often commensal. These interactions contribute to microbial balance and host homeostasis (Lof et al. 2017). *C. albicans*, for instance, commonly resides as a commensal in the gut, oral cavity, and vaginal tract without causing pathology. In these states, it engages in beneficial crosstalk, such as stimulating symbiotic bacteria like *Lactobacillus* to enhance the production of protective metabolites, thereby fostering a stable and cooperative relationship that contributes to microbial homeostasis and the proper education and function of the host immune system (Lof et al. 2017; Wang et al. 2023; Li et al. 2022).

**Figure 4:**
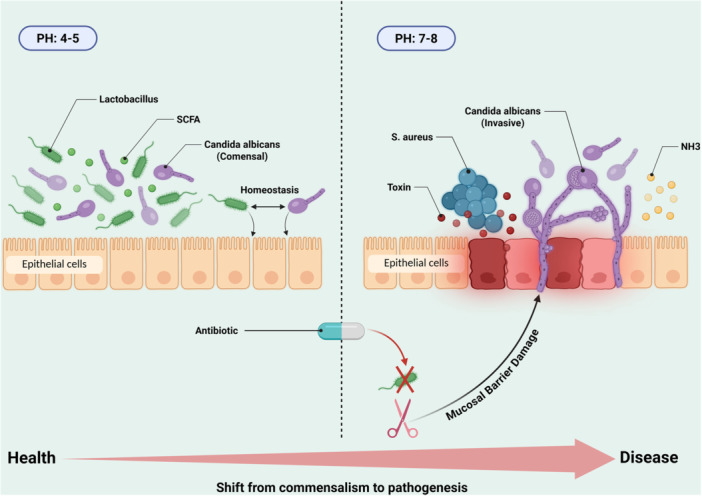
In the healthy state (left), a low pH environment (pH 4–5), maintained by commensal bacteria such as Lactobacillus and their metabolic products (short‐chain fatty acids, SCFAs), supports commensal growth of *C. albicans* and epithelial homeostasis. Disruption by factors such as antibiotic exposure (right) leads to depletion of protective bacteria and an increase in pH (7–8), triggering the transition of *C. albicans* to an invasive phenotype. Ammonia (NH_3_) production further elevates pH and promotes a microenvironment conducive to the proliferation of bacterial pathogens such as *S. aureus*, which release virulence factors. This cooperative interaction drives the shift from microbial homeostasis to polymicrobial disease.

However, this delicate balance is highly precarious. Host and environmental disruptions, including immunodeficiency, alterations in local pH or metabolism, and damage to mucosal barriers, can act as critical triggers, pivoting these symbiotic relationships toward frank pathogenicity (Faustino et al. 2025). This transition from commensal equilibrium to pathogenic dysbiosis, driven by environmental disruption and microbial cooperation, is schematically illustrated in Figure 4. A quintessential example is the antibiotic‐induced depletion of short‐chain fatty acid‐producing bacteria in the gut, which removes a key metabolic constraint on *Candida*, permitting its overgrowth and simultaneously creating an ecological opportunity for pathobionts like *E. faecalis* to expand (Zeise et al. 2021). The paradigm of ecological fitness is central to understanding this shift. In disease states, pathogens co‐opt their interactions to mutually enhance their survival and virulence. *C. albicans* can secrete metabolites that facilitate the growth of enterococci, while bacteria such as *S. aureus* exploit the tissue damage and inflammatory milieu created by fungal invasion to establish and expand their own colonies (Eichelberger et al. 2023; Hill and Round 2024). This cooperative virulence, achieved through division of labor and resource sharing, significantly increases the collective fitness of the microbial alliance at the direct expense of the host, leading to more severe and damaging infections.

A primary catalyst disrupting the commensal‐pathogen continuum is the use of broad‐spectrum antibiotics. By selectively eliminating susceptible members of the microbiota, these drugs create an ecological vacuum that is rapidly filled by resistant fungi and opportunistic bacterial pathogens, thereby dismantling the stable interactions that once maintained homeostasis. Human studies have demonstrated that treatment with amoxicillin‐clavulanate disrupts intestinal fungal‐bacterial networks and significantly elevates the risk of pathogenic colonization (Seelbinder et al. 2020).

In conclusion, the dynamic nature of fungal‐bacterial interactions defines a critical continuum between commensalism and pathogenesis. These relationships are fundamental to maintaining health under stable conditions but can rapidly transform into powerful drivers of disease when host‐microbe homeostasis is disrupted. A critical perspective reveals that the transition from symbiosis to dysbiosis is not merely a microbial phenomenon but a failure of host regulatory mechanisms. This understanding underscores the limitations of broad‐spectrum antimicrobials that further disrupt microbial ecology and highlights the urgent need for therapeutic strategies that support or restore beneficial microbial interactions rather than indiscriminately eliminating pathogens. The clinical challenge lies in identifying the precise tipping points where these interactions become pathogenic and developing interventions that can maintain or revert them to a commensal state.

## 7 Future Directions

Understanding fungal‐bacterial interactions in polymicrobial infections remains a rapidly evolving field with substantial translational potential. Future research should prioritize the development of high‐throughput screening platforms to systematically identify novel antimicrobial combinations capable of overcoming biofilm‐mediated resistance and counteracting antagonistic drug interactions. Additionally, host‐directed therapeutic strategies represent a promising frontier, particularly approaches focused on modulating macrophage polarization, enhancing neutrophil extracellular trap activity, or attenuating detrimental inflammatory cascades while preserving essential pathogen clearance mechanisms.

Advances in integrative multi‐omics technologies, including transcriptomics, metabolomics, and proteomics, offer unprecedented opportunities to decipher cross‐kingdom signaling networks and metabolic dependencies, enabling the identification of novel therapeutic targets for clinical intervention. Another crucial avenue involves microbiome engineering strategies, employing targeted probiotics or synthetic microbial consortia to disrupt pathogenic fungal‐bacterial alliances and restore mucosal homeostasis in affected patients.

There is an urgent need for well‐designed longitudinal clinical studies to establish causal relationships between specific microbial interactions and patient outcomes. Such research will be fundamental for developing precision medicine approaches tailored to at‐risk populations including intensive care unit patients, transplant recipients, and individuals with CF. These investigations should particularly focus on elucidating the temporal dynamics of microbial interactions and their impact on disease progression and treatment responses.

## 8 Conclusion

Fungal–bacterial interactions should no longer be viewed as incidental co‐isolation of microorganisms, but rather as dynamic, structured, and highly adaptive biological systems that directly shape infection outcomes. Evidence from clinically relevant pairs such as *C. albicans*–*S. aureus*, *P. aeruginosa*–*A. fumigatus*, and *Candida* spp.–*Enterobacteriaceae* demonstrates that cross‐kingdom interactions can profoundly enhance virulence, promote biofilm resilience, alter immune responses, and reduce antimicrobial efficacy.

This review highlights several conceptual advances beyond traditional pathogen‐centered frameworks. First, it emphasizes the importance of biophysical determinants such as biofilm viscoelasticity, matrix stiffness, and structural heterogeneity, which are increasingly recognized as key drivers of antimicrobial tolerance and represent underexplored therapeutic targets. Second, it consolidates current knowledge on diagnostic limitations in polymicrobial infections and evaluates emerging technologies, including metagenomics, MALDI‐TOF MS, and spatial imaging approaches, as essential tools for accurate detection and characterization of complex microbial communities.

Third, the review systematically discusses novel therapeutic strategies that move beyond conventional monotherapies. These include rational antibiotic–antifungal combinations, enzymatic and nanoparticle‐based biofilm disruption, bacteriophage therapy, QS interference, and metabolic targeting approaches designed to dismantle interspecies cooperation rather than merely eliminate individual pathogens. Importantly, it also underscores the growing relevance of host‐directed therapies aimed at modulating dysregulated immune responses that contribute significantly to disease severity in polymicrobial settings.

Finally, the concept of a polymicrobial continuum from commensalism to pathogenesis provides an integrative framework for understanding how microbial communities shift from symbiotic coexistence to disease‐driving consortia under host or environmental perturbations. Within this framework, disease is not simply the result of pathogen invasion, but rather a failure of ecological and immunological homeostasis that allows cooperative microbial networks to emerge and persist.

Despite significant progress, major challenges remain, particularly in translating mechanistic insights into clinically applicable diagnostic and therapeutic strategies. The absence of standardized approaches for polymicrobial infections, combined with the complexity of microbial interactions and biofilm‐associated resistance, continues to limit treatment efficacy in high‐risk patient populations.

Addressing these challenges will require a shift toward interdisciplinary and systems‐level approaches that integrate microbiology, immunology, biophysics, and clinical medicine. Future advances will depend on the development of rapid multiplex diagnostics, biofilm‐aware susceptibility testing, and personalized therapeutic strategies informed by microbial ecology and host response profiling. Ultimately, recognizing polymicrobial infections as coordinated and adaptive biological systems represents a critical paradigm shift that will shape the next generation of diagnostic and therapeutic innovations in infectious diseases.

## Author Contributions

**Mohammad Javad Roustaye Gourabi:** writing – original draft, writing – review and editing. **Masoud Kargar:** writing – original draft,visualization, investigation. **Atefeh Kamali:** writing – original draft, writing – review and editing. **Javad Yasbolaghi Sharahi:** conceptualization, supervision, investigation, writing – original draft, writing – review and editing, project administration.

## Funding

The authors have nothing to report.

## Ethics Statement

The authors have nothing to report.

## Conflicts of Interest

The authors declare no conflicts of interest.

## Data Availability

Data sharing not applicable to this article as no datasets were generated or analyzed during the current study.
